# ζ-Glycine: insight into the mechanism of a polymorphic phase transition

**DOI:** 10.1107/S205225251701096X

**Published:** 2017-09-01

**Authors:** Craig L. Bull, Giles Flowitt-Hill, Stefano de Gironcoli, Emine Küçükbenli, Simon Parsons, Cong Huy Pham, Helen Y. Playford, Matthew G. Tucker

**Affiliations:** a ISIS Facility, Rutherford–Appleton Laboratory, Harwell Science and Innovation Campus, Didcot, Oxfordshire OX11 0QX, UK; b School of Chemistry and Centre for Science at Extreme Conditions, The University of Edinburgh, King’s Buildings, W. Mains Road, Edinburgh EH9 3FJ, UK; c Scuola Internazionale Superiore di Studi Avanzati, Via Bonomea 265, Trieste 34136, Italy; d Department of Chemistry and Biochemistry, University of California San Diego, La Jolla, CA 92093, USA; e Oak Ridge National Laboratory, PO Box 2008, Oak Ridge, TN 37831, USA

**Keywords:** amino acids, crystal structure prediction, polymorphism, neutron diffraction, phase transitions, crystallization under non-ambient conditions

## Abstract

Glycine is the simplest and most polymorphic amino acid, with five phases having been structurally characterized at atmospheric or high pressure. A sixth form, the elusive ζ phase, was discovered over a decade ago as a short-lived intermediate which formed as the high-pressure ∊ phase transformed to the γ form on decompression. However, its structure has remained unsolved. We now report the structure of the ζ phase, which was trapped at 100 K enabling neutron powder diffraction data to be obtained. The structure was solved using the results of a crystal structure prediction procedure based on fully *ab initio* energy calculations combined with a genetic algorithm for searching phase space. We show that the fate of ζ-glycine depends on its thermal history: although at room temperature it transforms back to the γ phase, warming the sample from 100 K to room temperature yielded β-glycine, the least stable of the known ambient-pressure polymorphs.

## Abstract 2

The structure of the ζ polymorph of glycine has been solved by a combination of crystal structure prediction and neutron powder diffraction, where the elusive phase was trapped at low temperature. At room temperature ζ-glycine is a short-lived intermediate in the pressure-driven transition between the ∊ and γ polymorphs; by contrast at low temperature it undergoes the first observed transformation into the metastable β polymorph.

## 1. Introduction   

Glycine, the simplest amino acid, exhibits greater phase diversity than any of the other naturally occurring amino acids. All phases consist of the zwitterionic form ^+^H_3_N—CH_2_—CO_2_^−^ and three polymorphs are known under ambient conditions. The α and β forms are both monoclinic (space groups *P*2_1_/*n* and *P*2_1_, respectively), with crystal structures composed of hydrogen-bonded layers. The γ form is trigonal (*P*3_1_) and displays a three-dimensional hydrogen-bonded network featuring chains disposed about 3_1_ screw axes. Of the three polymorphs, γ-glycine is the most thermodynamically stable at room temperature (Perlovich *et al.*, 2001 ▸).

The effect of pressure on the polymorphs of glycine has been extensively investigated. Although α-glycine persists to 23 GPa (Murli *et al.*, 2003 ▸), β-glycine undergoes a phase transition at only 0.8 GPa to the δ phase (*P*2_1_/*n*; Dawson *et al.*, 2005 ▸; Goryainov *et al.*, 2005 ▸). This transition is displacive and fully reversible as a result of the topological similarity of the β and δ phases, consisting of a concerted inversion of the O—C—C—N torsion angles in half the molecules which otherwise retain their positions and orientations. Application of pressure to γ-glycine leads to ∊-glycine (*Pn*; Boldyreva *et al.*, 2005 ▸; Dawson *et al.*, 2005 ▸; Moggach *et al.*, 2015 ▸). The structure changes substantially, the threefold helices being replaced by a layered structure. The transition is sluggish and reconstructive, but is complete at between 4 and 5 GPa. Note that some authors refer to the δ and ∊ phases as β′ and δ, respectively.

The γ- to ∊-glycine transition shows considerable hysteresis and the ∊ phase persists on decompression to 0.28 GPa (Moggach *et al.*, 2015 ▸). Complete release of applied load yields yet another phase, ζ-glycine, which exists for as little as 30 min at room temperature before transforming back to the γ phase (Bordallo *et al.*, 2008 ▸; Goryainov *et al.*, 2006 ▸; Moggach *et al.*, 2015 ▸). The ζ phase, which has been characterized by X-ray powder diffraction and Raman spectroscopy, is only observed upon decompression of the ∊ form; application of pressure to the γ phase yields the ∊ phase directly.

Although the ζ phase was first identified over a decade ago, its structure is unknown. On the basis of Raman data it has been proposed to have a layered structure similar to all the other phases except for γ-glycine (Bordallo *et al.*, 2008 ▸). In this paper we describe the solution of the crystal structure of ζ-glycine using high-quality neutron powder diffraction measurements in combination with crystal structure prediction based on fully first-principles total energy calculations and an improved genetic search algorithm for searching the phase space.

## 2. Experimental   

### 2.1. Neutron powder diffraction   

A sample of γ-glycine-d_5_ (CDN Isotopes, Canada) was obtained by non-photochemical laser-induced nucleation of a supersaturated solution in D_2_O (Zaccaro *et al.*, 2001 ▸). Colourless needle-like crystals were allowed to grow for 2 h at room temperature and then lightly crushed to form a polycrystalline sample. Pressure was applied to the sample using a Paris–Edinburgh press modified for use at low temperature, utilizing zirconia-toughened alumina anvils. The sample was enclosed in a null-scattering TiZr encapsulated gasket. A mixture of deuterated methanol and ethanol (4:1 *v*/*v*) was used as a pressure transmitting medium with a lead pellet as pressure marker. Diffraction data were obtained on the PEARL instrument at ISIS (Bull *et al.*, 2016 ▸) and the resulting diffaction corrected for anvil attenuation. The sample was compressed to 5.56 GPa to yield a phase-pure sample of ∊-glycine. The temperature was then rapidly lowered to 200 K and the load reduced to 0 tonnes. Then the temperature was further reduced to 100 K. Neutron powder diffraction data were collected over the following 5 h. Following data collection, the sample was warmed to room temperature while monitoring the diffraction pattern. Further experimental details are available in the supporting information.

### 2.2. Crystal structure prediction   

The evolutionary algorithm as implemented in the *USPEX* package (Lyakhov *et al.*, 2013 ▸) was used to search for the low-energy structures of glycine with *Z* = 2, 3 or 4 molecules in the unit cell. In the first generation 30 structures were created randomly. The energetically least favourable 20% of the population was discarded. A fingerprint analysis of the remaining structures was performed (Oganov & Valle, 2009 ▸). The structures whose fingerprint was within an adimensional cosine distance of 0.01 from any lower energy structure were also discarded. The remaining structures were then eligible as parents and allowed to ‘procreate’. Thirty new structures of the next generation were created from the parents through the following operations: heredity (crossover of two structures) (40%), soft mutation (translation and rotation based on an estimate of soft vibrational modes) (20%), rotation of the molecule (20%) and random structure generation (20%). In addition, the three best parents were cloned directly to the next generation. In all simulations, the maximum number of generations was 20.

### 2.3. *Ab initio* calculations   

For every structure generated by *USPEX*, the geometry and cell relaxation were calculated using a van der Waals density functional (vdW-DF) as implemented in the *QUANTUM ESPRESSO* package (Giannozzi *et al.*, 2009 ▸). A kinetic energy cutoff of 80 Ryd and a charge density cutoff of 560 Ryd were used. The Brillouin zone sampling resolution was gradually increased over three steps during relaxation, from 2π × 0.12 Å^−1^ to 2π × 0.10 Å^−1^ and finally 2π × 0.08 Å^−1^. Energies and geometries from the last step with the densest *k*-point grid were used throughout the study. Projector augmented wave (PAW) pseudopotentials were taken from the *PSLibrary v0.1* (Küçükbenli *et al.*, 2014 ▸). By using this setup all structures were fully relaxed within a convergence of less than 0.1 mRyd for the absolute total energy per molecule, 0.5 mRyd a.u.^−1^ for the forces on the atoms and less than 0.005 GPa for the pressure obtained from the stress tensor.

## 3. Results   

### 3.1. Crystal structure prediction   

Conventional methods for indexing and solving the structure of ζ-glycine failed to yield models which reproduced the experimental data. However, independently of the diffraction experiments, theoretical work had been carried out to assess the predictive power of a newly developed crystal structure prediction (CSP) workflow using glycine as the test case. Although a number of CSP surveys of glycine have been described (Zhu *et al.*, 2012 ▸; Lund *et al.*, 2015 ▸; Chisholm *et al.*, 2005 ▸), none has so far identified candidates which fit the experimental data for ζ-glycine, though a combined CSP–powder diffraction study has very recently yielded the crystal structure of glycine dihydrate (Xu *et al.*, 2017 ▸).

A critical component of CSP is the method of evaluating the lattice energies of candidate phases. This determines both energy ranking and the direction of the phase-space search. The highest quality energies are obtained using first-principles calculations based on periodic density functional theory (DFT) (Hohenberg & Kohn, 1964 ▸; Reilly *et al.*, 2016 ▸), but despite some spectacular successes (Woodley & Catlow, 2008 ▸) molecular crystals are challenging because of the difficulties in accounting for dispersion interactions (Reilly *et al.*, 2016 ▸).

**Figure 1:**
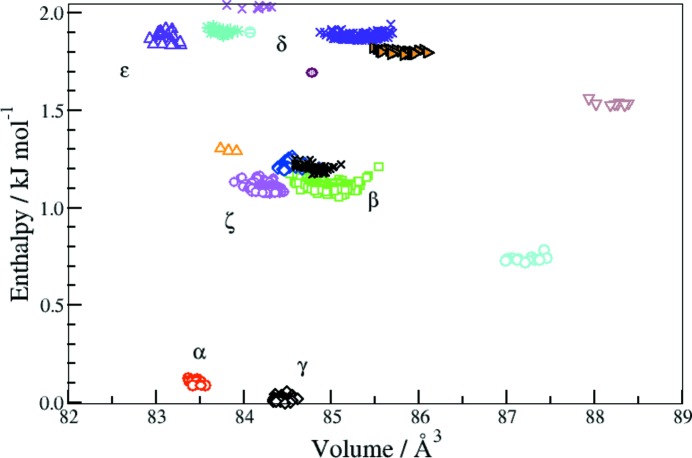
Enthalpy *versus* volume distribution for all the structures obtained *via ab initio* crystal structure search within the lowest 2 kJ mol^−1^ range of the most stable γ phase. Crowding around each polymorph indicates multiple encounters with the same phase during the phase-space exploration.

In this study energies were calculated (*QUANTUM ESPRESSO*; Giannozzi *et al.*, 2009 ▸) with a recently developed functional (vdW-DF; Berland *et al.*, 2015 ▸) which takes dispersion into account *via* a non-local functional of the overall charge density. The method yields reliable energy differences between phases of molecular materials (Sabatini *et al.*, 2012 ▸), including the ambient-pressure forms of glycine, without suffering from the transferability problems of empirical methods. The combination of this method for energy evaluation with the genetic algorithm for searching phase space (*USPEX*; Lyakhov *et al.*, 2013 ▸) assuming two, three or four molecules per unit cell yielded a very rich series of hypothetical structures for glycine (Fig. 1 ▸). Fuller details are available in the supporting information.

In addition to all the experimentally observed phases of glycine, the CSP analysis predicted several new phases within 2 kJ mol^−1^ of the most stable (γ) phase. In particular, the survey had identified a potential new polymorph in *P*1 with one molecule per unit cell (*Z* = 1; *a* = 5.0168, *b* = 4.7491 and *c* = 4.0593 Å; α = 95.8383, β = 105.6522 and γ = 64.8726°) that was very close in energy to the known β polymorph. This was proposed as ζ-glycine based on the agreement between previous mixed-phase X-ray diffraction experiments (Boldyreva *et al.*, 2005 ▸) and theoretical assessment (see supporting information). The structural parameters of the new phase, which had been predicted completely *ab initio*, were found to form a suitable starting model for refinement of the structure against the neutron diffraction data.

### 3.2. Neutron powder diffraction   

Neutron powder diffraction data suitable for structure solution were collected for ζ-glycine by trapping the phase at 100 K, extending its lifetime for long enough to collect a high-quality diffraction pattern.

**Figure 2:**
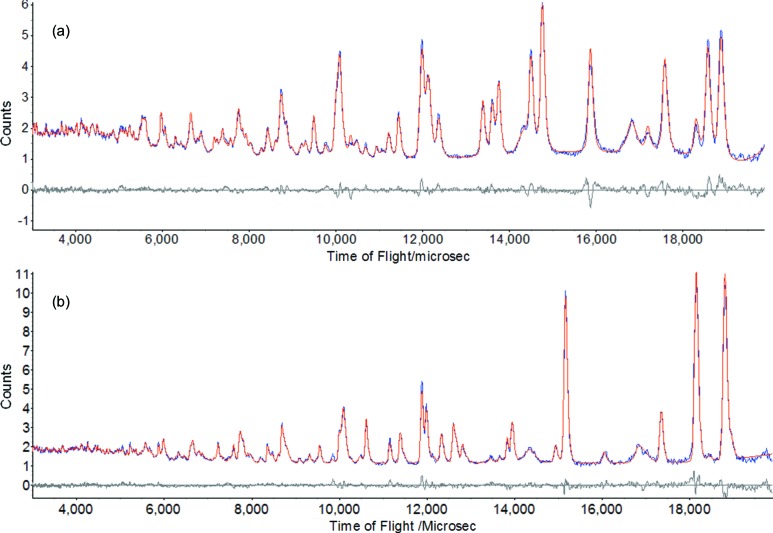
(*a*) Rietveld fit of the neutron powder diffraction pattern of ζ-glycine at 100 K (blue = observed, red = calculated). In addition to the peaks from ζ-glycine, the pattern also shows the presence of residual ∊- and a trace of γ-glycine. Other peaks arise from the sample environment, namely the lead pressure marker and the Al_2_O_3_ and ZrO_2_ components of the anvils of the pressure cell. (*b*) Rietveld fit of the neutron powder diffraction pattern of β-glycine (contaminated with ζ- and a trace of γ-glycine) at 290 K. A 1 Å *d* spacing approximates to 4837 µs in time-of-flight.

The powder diffraction data were indexed using the unit-cell dimensions obtained in the structure prediction multiplied by a factor of 0.98. This accounts for the tendency of the method used for CSP to overestimate slightly the unit-cell dimensions of other glycine polymorphs (Table S1). Pawley fitting yielded the following cell dimensions for ζ-glycine: *a* = 4.9307 (3), *b* = 4.54798 (4) and *c* = 3.9191 (3) Å, α = 95.550 (5), β = 105.250 (5) and γ = 64.938 (6)°, and *V* = 76.797 (11) Å^3^. These values can be transformed to a body-centred triclinic setting containing two molecules per unit cell, which clarifies the structural relationship of ζ-glycine with the other phases. These transformed cell dimensions are compared with those of the other *Z* = 2 phases in Table 1 ▸. The predicted crystal structure of ζ-glycine was refined using Rietveld methods (*TOPAS-Academic*; Coelho, 2015 ▸). The final fit is shown in Fig. 2 ▸(*a*).

When the sample of ζ-glycine prepared in this study was warmed from 100 K, the peaks due to the residual ∊ phase were first seen to disappear at 250 K. At 290 K the peaks due to ζ-glycine began to diminish, to yield not the expected γ phase but rather the β phase (Fig. 2 ▸ *b*). Persistence of the ζ polymorph after the disappearance of the ∊ polymorph shows that the transition to β-glycine is from ζ-glycine rather than ∊-glycine; the initial disappearance of ∊-glycine is the continuation of the original ∊ to ζ transition halted by the temperature decrease.

## 4. Discussion   

### 4.1. Structural relationships and phase stability   

The data in Table 1 ▸ show that the unit-cell dimensions of the β, ∊ and ζ polymorphs are very closely related to each other. The structures, along with that of γ-glycine, are shown in Figs. S13–S16. The β, ∊ and ζ phases are layered structures in which the molecules in the unit cell reside near positions equivalent to 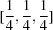 and 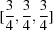 (Table S4). A crystallographic fractional coordinate of 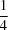 can be transformed to 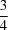 either by adding 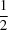 or by inverting it and adding 1. The β, ∊ and ζ polymorphs can simply be viewed as the result of various combinations of these operations along the *x*, *y* and *z* directions of the unit cell. In the ζ phase the relationship is *x* + 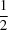, *y* + 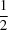, *z* + 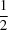 (*I*-centring); in the ∊ phase it is *x* + 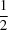, −*y* + 1, *z* + 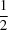 (an *n*-glide plane), while in the β phase it is −*x* + 1, *y* + 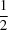, −*z* + 1 (a 2_1_ screw axis).

In the case of the β and ζ phases the symmetry operations preserve conformation, and this is why the cell dimensions of these two phases are so similar. In ∊-glycine the two zwitterionic molecules are related by an *n*-glide and have opposite conformations, the N1—C2—C1—O2 torsion angles being ±16.0 (5)°. In ζ-glycine the torsion angle increases to 35.1 (4)° and all the molecules have the same conformation. The ∊- to ζ-glycine transition can thus be viewed as a switch of conformation of half the molecules in the structure, and in this regard it is similar to the β- to δ-glycine transition at 0.8 GPa. Although γ-glycine shares the polarity of the ∊ and ζ phases along **c**, and since its space group is chiral all three molecules in the unit cell share the same conformation [τ(N1—C2—C1—O2) = 15.6 (1)°; Kvick *et al.*, 1980 ▸], its structure consists of a three-dimensional hydrogen-bonded network rather than layers.

Symmetry-adapted perturbation theory (*PSI4* software; Turney *et al.*, 2012 ▸) and *PIXEL* calculations (Gavezzotti, 2005 ▸) (see supporting information) both indicate that the strongest intermolecular interaction in ζ-glycine, with an energy in excess of −100 kJ mol^−1^, is a ‘head-to-tail’ hydrogen bond formed between ammonium and carboxyl­ate groups. Repetition of this interaction builds a chain which runs along the **c** direction of the crystal structure. This same chain is present in all six known polymorphs, and the phases differ in the way these chains interact.

**Figure 3:**
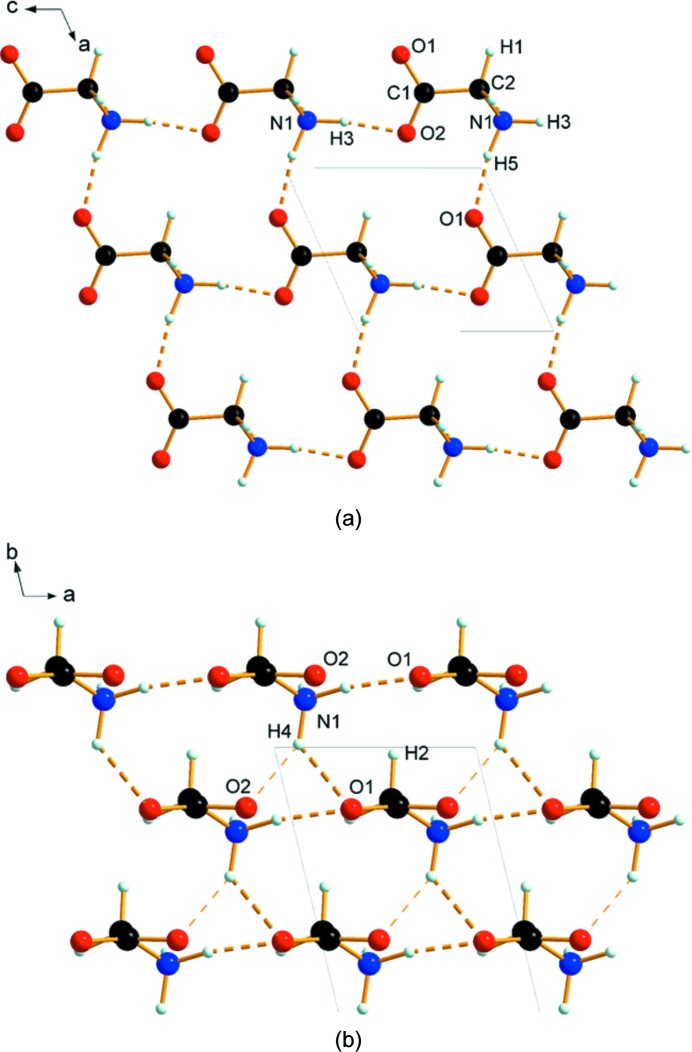
Intermolecular interactions in ζ-glycine. (*a*) Layers formed in the *ac* plane, viewed along **b**. (*b*) Stacking of the layers, viewed along **c**.

In ζ-glycine the chains are arranged in the *ac* plane, related by lattice repeats along **a**, forming a layer (Fig. 3 ▸ *a*). This layer motif confirms Boldyreva’s conclusion regarding the layered nature of the ζ phase made on the basis of vibrational spectroscopy (Bordallo *et al.*, 2008 ▸). The layers are very similar, both in terms of geometry and in the molecule–molecule energies, to those seen for the ∊ phase. The intermolecular contact energy across the N1—H5⋯O1 hydrogen bonds (−26 kJ mol^−1^) is only about a quarter of that of the head-to-tail linkages, a result of the repulsive influence of neighbouring carboxyl­ate groups.

In the ζ phase all layers are connected through equivalent N1—H4⋯O contacts (Fig. 3 ▸ *b*). The layer separation is *b*/2 = 3.14 Å. Although the intermolecular contact energy for the pairs of molecules linked by N1—H4⋯O1 contacts (−59.4 kJ mol^−1^) is equivalent to a strong hydrogen bond, the geometry of the contact deviates markedly from linearity [∠N1—H4⋯O1 = 121.1 (10)°] with a relatively long H⋯O distance [2.274 (5) Å], and the interaction is more plausibly classified as a simple electrostatic interaction involving ammonium and carboxylate groups.

The relative energies (in kJ mol^−1^) of the six phases of glycine, calculated using DFT and including the approximation for van der Waals interactions described above, are: γ 0, α 0.084, β 1.055, ζ 1.070, ∊ 1.832 and δ 2.019. The energies refer to geometry-optimized structures at 0 K and 0 Pa. The ordering of the α, β and γ phases agrees with previous work (Marom *et al.*, 2013 ▸; Perlovich *et al.*, 2001 ▸; Sabatini *et al.*, 2012 ▸), while the total range, which spans 2 kJ mol^−1^, agrees with Hunter’s recent results on typical polymorph energy differences (Hunter & Prohens, 2017 ▸). The ζ phase is marginally less stable than the previously known ambient-pressure phases, but more stable than either of the high-pressure phases.

### 4.2. The fate of ζ-glycine on warming   

Previous work (Boldyreva *et al.*, 2005 ▸; Bordallo *et al.*, 2008 ▸; Goryainov *et al.*, 2006 ▸; Moggach *et al.*, 2015 ▸) using X-ray and neutron powder diffraction and vibrational spectroscopy has shown that ζ-glycine transforms spontaneously to the γ phase at room temperature. By contrast, in the present investigation, warming the sample from 100 K to room temperature yielded β-glycine rather than the expected γ phase. The thermal history of the samples used in this and previous work were different: in previous studies all manipulations were carried out at room temperature, whereas here the sample had been cooled. Nevertheless the difference in behaviour is surprising, and this is the first time, to our knowledge, that a transition to the metastable β phase of glycine has been observed. Similar sensitivity of phase formation to thermal history has been observed, for example in paracetamol (Qi *et al.*, 2008 ▸; Rossi *et al.*, 2003 ▸), while in glycine itself the temperature of the thermal γ-to-α phase transition can be increased by *ca* 10 K by annealing the sample (Perlovich *et al.*, 2001 ▸).

Over the course of the ∊–ζ–γ transition (see the movie in the supporting information), the layers of ∊-glycine first slide over one another with small molecular rotations to give ζ-glycine. Larger reorientations that disrupt the layer structure are needed as the system transforms to the γ phase.

As discussed above, the β and ζ phases are closely related. Both consist of chains composed of head-to-tail N1—H3⋯O2 hydrogen bonds which are connected into a layer *via* N1—H5⋯O1 hydrogen bonds. The difference between the phases is that the *c* axis is a polar direction in the ζ phase but not in the β phase. Therefore, in the ζ–β transition (see the movie in the supporting information) the layer structure is retained but a rotation of layers with respect to one another is needed. The lack of a strong directional preference in the electrostatic contacts between the layers (see above) may explain why such phase transitions involving rearrangements of layer stacking can occur.

The structure of ζ-glycine is significant because the phase is akin to a supramolecular reactive intermediate, providing insight into the mechanism of solid-state phase transitions. The results presented here, along with those of previous studies (Boldyreva *et al.*, 2005 ▸; Bordallo *et al.*, 2008 ▸; Goryainov *et al.*, 2006 ▸; Moggach *et al.*, 2015 ▸), indicate that two transformation pathways are available to ζ-glycine, one leading to the β phase and the other to the γ phase. One possibility is that the activation barrier to the β phase is lower, and it is possible that seeds of this phase begin to form at low temperature. In this interpretation, at ambient temperature there is enough thermal energy to form the thermodynamically more stable γ phase. Alternatively, the topological similarity of the ∊ and γ phases, in which all the head-to-tail chains have the same polarity, may enable a residual amount of the high-energy ∊ phase to seed the formation of γ-glycine. Further theoretical modelling and experimental investigations would provide valuable insight into the physical source of the difference between the low- and ambient-temperature behaviour.

## 5. Related literature   

For additional literature relating to the supporting information, see Brandenburg (2004 ▸), Destro *et al.* (2000 ▸), Fortes *et al.* (2007 ▸, 2012 ▸), Gavezzotti (2011 ▸), Hohenstein & Sherrill (2010*a* ▸,*b* ▸, 2012 ▸), Jeziorski *et al.* (1994 ▸), Frisch *et al.* (2009 ▸), Macrae *et al.* (2008 ▸), Sheldrick (2001 ▸), Spek (2003 ▸) and Stone (2013 ▸).

## Supplementary Material

Crystal structure: contains datablock(s) zeta_glycine_100K, epsilon_glycine_100K, profile_100K, beta_glycine_290K, zeta_glycine_290K, profile_RT. DOI: 10.1107/S205225251701096X/lq5008sup1.cif

Further details relating to the crystal structure prediction and crystallographic structure refinements. Figures showing the structures of all the phases discussed are given, along with an energy analysis of intermolecular contacts in the epsilon and zeta phases.. DOI: 10.1107/S205225251701096X/lq5008sup2.pdf

Movie (animated gif, ezb.gif). DOI: 10.1107/S205225251701096X/lq5008sup3.gif

CCDC references: 1541927, 1541928, 1541929, 1541930

## Figures and Tables

**Table 1:** Cell dimensions of β-, ∊- and ζ-glycine In all cases *Z* = 2. All values are from this work.

	∊ (100 K)	ζ (100 K)	β (298 K)	ζ (298 K)
Symmetry	*Pn*	*I*1	*P*2_1_	*I*1
*a* (Å)	5.0230 (4)	5.1000 (4)	5.0907 (2)	5.1029 (16)
*b* (Å)	5.9846 (4)	6.2850 (3)	6.25954 (16)	6.3450 (12)
*c* (Å)	5.4946 (5)	5.4295 (3)	5.38710 (19)	5.4331 (18)
α (°)	90	85.815 (5)	90	85.91 (3)
β (°)	114.654 (8)	114.456 (5)	113.261 (4)	114.26 (3)
γ (°)	90	104.136 (5)	90	103.55 (3)
Volume (Å^3^)	150.12 (2)	153.545 (17)	157.710 (10)	155.85 (9)
