# Supplementary material for: ζ-Glycine: insight into the mechanism of a polymorphic phase transition
Source: IUCrJ. 2017 Sep 1;4(Pt 5):569–74. doi: 10.1107/S205225251701096X (PMC5619850; doi:10.1107/S205225251701096X)
Supplement: Supplementary file 2 [file m-04-00569-sup2.pdf]

# IUCrJ

**Volume 4 (2017)**


**Supporting information for article:**

## ζ-Glycine: Insight into the Mechanism of a Polymorphic Phase Transition

**Craig L. Bull, Giles Flowitt-Hill, Stefano de Gironcoli, Emine Küçükbenli, Simon Parsons, Cong Huy Pham, Helen Y. Playford and Matthew G. Tucker**

# Supplementary Material

## ζ-Glycine: Insight into the Mechanism of a Polymorphic Phase Transition

Authors

**Craig L. Bull[a], Giles Flowitt-Hill[ab], Stefano de Gironcoli[c]*, Emine Küçükbenli[c]*, Simon Parsons[b]*, Cong Huy Pham[cd], Helen Y. Playford[a] and Matthew G. Tucker[ae]**

[a]ISIS Facility, Rutherford-Appleton Laboratory, Harwell Science and Innovation Campus, Didcot, Oxfordshire, OX11 0QX, UK
[b]School of Chemistry and Centre for Science at Extreme Conditions, The University of Edinburgh, King's Buildings, W. Mains Road, Edinburgh, EH9 3FJ, UK
[c]Scuola Internazionale Superiore di Studi Avanzati, Via Bonomea 265, 34136 Trieste, Italy
[d]Department of Chemistry and Biochemistry, University of California San Diego, La Jolla, California, 92093, USA
[e] Oak Ridge National Laboratory, PO Box 2008, Oak Ridge, Tennessee, 37831, USA

Correspondence email: degironc@sissa.it; kucukben@gmail.com; S.Parsons@ed.ac.uk

**Funding information** Science and Technology Facilities Council/ISIS (award No. RB1520301); Engineering and Physical Sciences Research Council (award No. DTA); SISSA, CINECA and PRACE (award No. 2011050736).

**S1. Experimental Procedures**

*S1.1 Ab initio Crystal Structure Prediction:*

The CSP procedure starts with a population of random structures in the first generation which then evolves such that only the thermodynamically most stable members are allowed to 'procreate', so that each generation inherits energy-reducing geometric patterns from previous ones. The procreation operations are cross-overs of parent structures, and "mutations" that vary the molecular position and orientation. To guarantee the diversity of the population throughout the evolution, new random structures are added at each generation, while new members that are too similar to previously sampled ancestor structures are prohibited from becoming parents for future generations. In this study the searches of phase space assumed 2, 3 and 4 glycine molecules per unit cell

The *ab initio* calculation of energy and geometry optimization used during the search procedure, though accurate, comes with increased computational cost. Since this would limit the explored phase space several strategies were employed to accelerate the search. These included retention of low-energy candidates over the evolution of several generations, and the use of supramolecular arrangements seen in the known phases in the search, i.e. using glycine dimer of the α polymorph as the search unit. We also employed a new strategy of biasing the space group of the randomly chosen members towards those observed in naturally occurring molecular crystals.

The following lists the enthalpy vs. volume diagrams obtained throughout the phase space explorations during crystal structure prediction (CSP) in this work. Each diagram represents a different search strategy and summarizes the outcome. Only the structures within the first 4 kJ mol$^{-1}$ are shown.

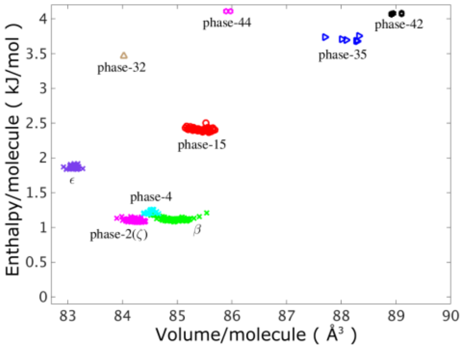


**Figure S1.** CSP for 2 molecule/cell without using any experimental information on the known crystal structures of the molecule. The simulation is run for 18 generations with settings described in the main text. Phases are labelled according to their enthalpy ranking in the whole search.

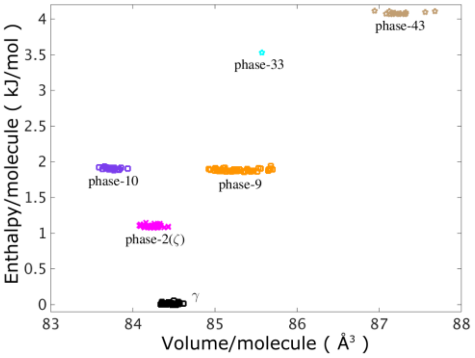


**Figure S2.** CSP for 3 molecule/cell without using any experimental information on the known crystal structures of the molecule. The simulation is run for 15 generations with settings described in the main text. Phases are labelled according to their enthalpy ranking in the whole search.

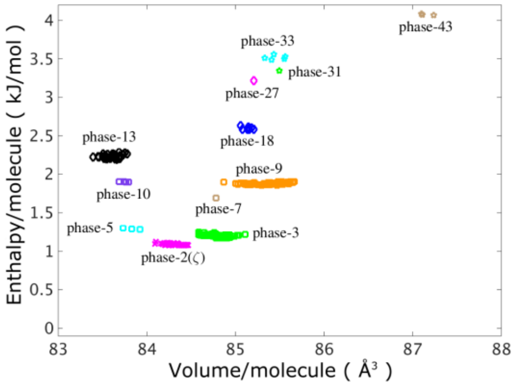


**Figure S3.** CSP for 3 molecule/cell in which the randomly generated structures were biased to represent the space group distribution of the known organic crystal structure database: $P2_1/c$ 36.59%, P1bar 16.92%, $P2_12_12_1$ 11.00%, C2/c 6.95%, $P2_1$ 6.35% Pbca 4.24%. The simulation is run for 20 generations with settings described in the main text. Phases are labelled according to their enthalpy ranking in the whole search. This search fails to find the most stable γ-phase of glycine in 20 generations.

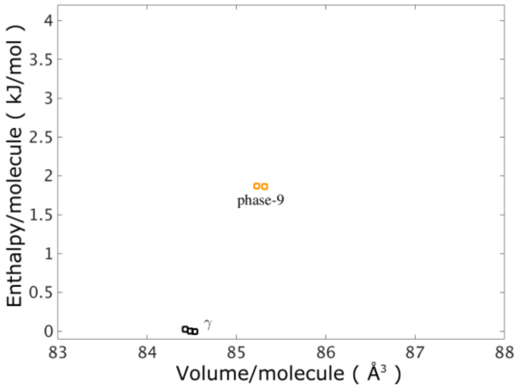


**Figure S4.** CSP for 3 molecule/cell in which the randomly generated structures were biased to represent the space group distribution of the known organic crystal structure database: $P2_1/c$ 36.59%, P1bar 16.92%, $P2_12_12_1$ 11.00%, C2/c 6.95%, $P2_1$ 6.35% Pbca 4.24%. The simulation is run for 2 generations with settings described in the main text. Phases are labelled according to their enthalpy ranking in the whole search. This search identifies the most stable γ-phase in the first generation.

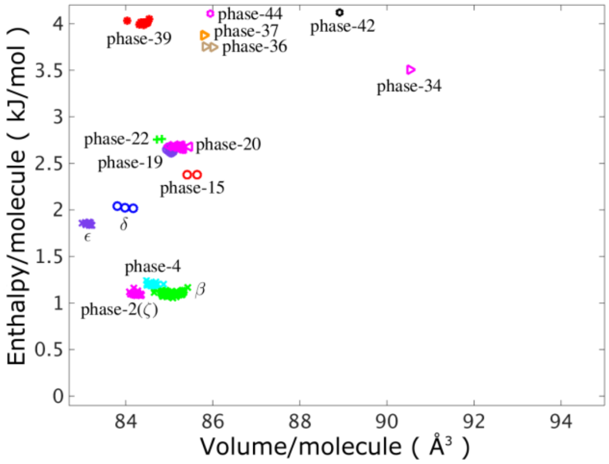


**Figure S5.** CSP for 4 molecule/cell without using any experimental information on the known crystal structures of the molecule. The simulation is run for 20 generations with settings described in the main text. Phases are labelled according to their enthalpy ranking in the whole search. This simulation fails to find α-phase within 20 generations.

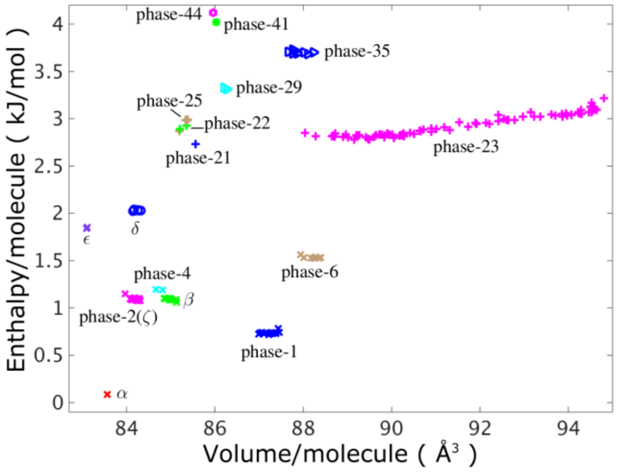


**Figure S6.** CSP for 4 molecule/cell in which the randomly generated structures were biased to represent the space group distribution of the known organic crystal structure database: $P2_1/c$ 36.59%, P1bar 16.92%, $P2_12_12_1$

11.00%, C2/c 6.95%, $P2_1$ 6.35% Pbca 4.24%. The simulation is run for 16 generations with settings described in the main text. Phases are labelled according to their enthalpy ranking in the whole search. This search identifies the α-phase in the first generation.

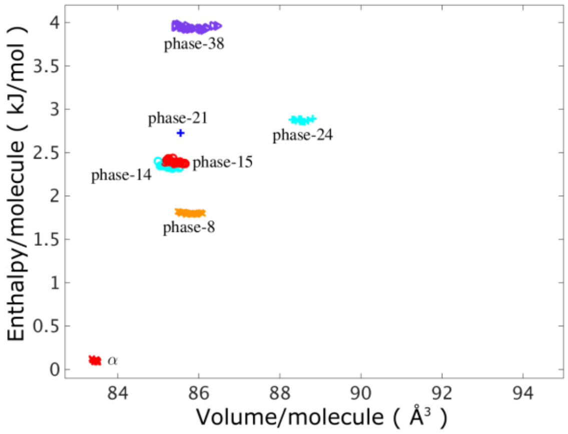


**Figure S7.** CSP for 4 molecule/cell using the glycine dimer seen in the α-phase as the building block of the crystal structure, instead of the single molecule as done in all other cases. The simulation is run for 7 generations with settings described in the main text. Phases are labelled according to their enthalpy ranking in the whole search. Note that in this search there are new phases that were not identified using 4 single molecules per cell, hinting at the vastness of the phase space and poor connectivity between enthalpy valleys.

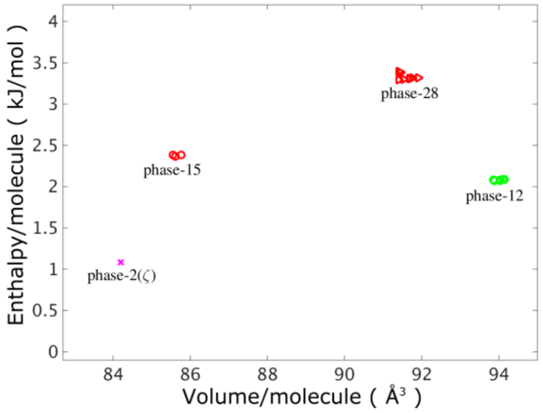


**Figure S8.** CSP for 4 molecule/cell using an increased similarity penalty between already encountered structures and the new ones proposed during evolution such that structures that are more different from one another are produced. The simulation is run for 5 generations with settings described in the main text.  Phases are labelled according to their enthalpy ranking in the whole search.

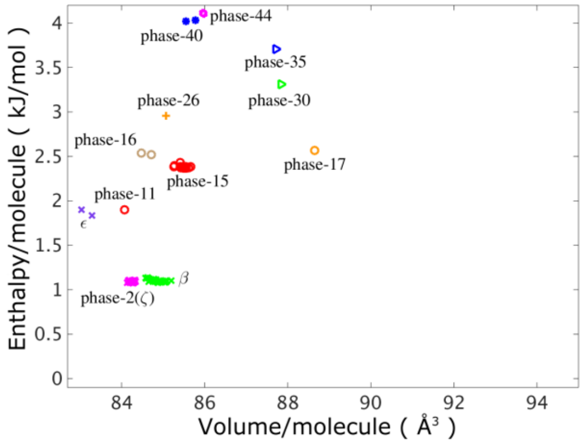


**Figure S9.** CSP for 4 molecule/cell in which the randomly generated structures are strictly taken from the P21/n space group that α-phase belongs to. The simulation is run for 14 generations with settings described in the main text. Phases are labelled according to their enthalpy ranking in the whole search. Note that this search does not find the α-phase within 14 generations.

*S1.2 Theoretical assignment of the ζ-glycine phase:*

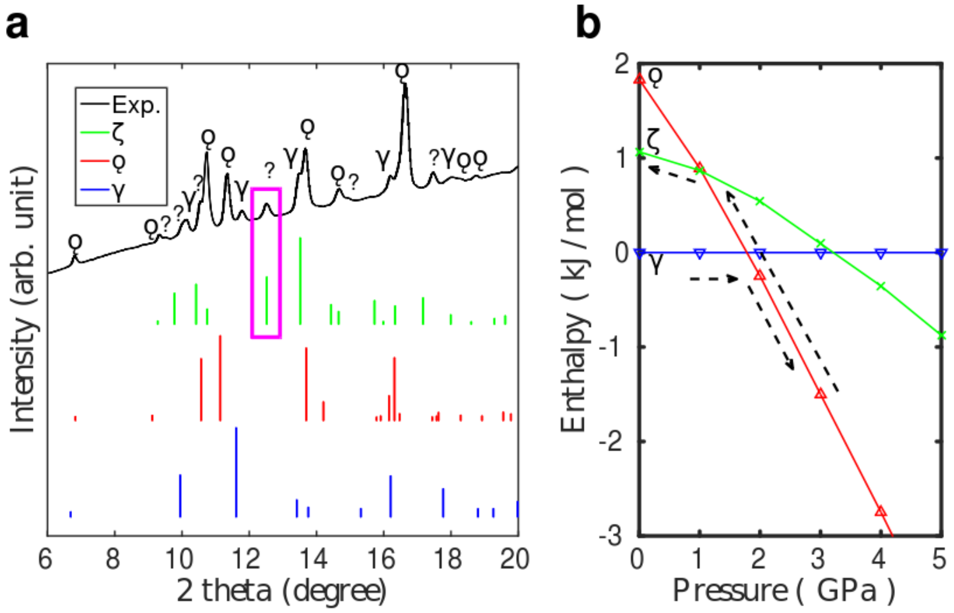


**Figure S10.** Theoretical assignment of the ζ-phase is done by **a)** comparison of simulated X-ray diffraction pattern for ε- γ- and ζ-glycine at 2GPa, with experimental data taken from Ref.4a of main text at 0.2 GPa. The XRD of proposed ζ-glycine can explain most of the unassigned peaks that were marked in the experimental spectrum. The theoretical spectra are calculated at higher pressure to offset the overestimation of ground state volumes. b) The enthalpy per molecule as a function of pressure for ε-glycine and ζ-glycine with respect to the γ phase up to 5 GPa is given. The black arrows indicate the phase transitions observed experimentally at room temperature: Under pressure the γ phase undergoes a transition to ε-glycine and upon decompression of ε-glycine, ζ-glycine is observed before the transition is completed to γ-glycine again.

Table S1 compares the energies and experimental and computed volumes of the α-ζ phases.

| **Table S1.** Enthalpy difference with respect to γ-phase and volume obtained with vdW-DF method compared to experimentally obtained values from literature. All values are per molecule. | | | |
|---|---|---|---|
| Polymorph | Delta Ent. (kJ mol$^{-1}$) | Volume (Å$^3$) | Exp. volume (Å$^3$) |
| **γ** | 0 | 84.477 | 76.9[a] |
| **α** | 0.084 | 83.515 | 75.8[b] |
| **β** | 1.055 | 85.083 | 79.2[c],78.85[e] |
| **ζ** | 1.070 | 84.305 | 76.8[d],77.9[e] |
| **ε** | 1.832 | 83.172 | 75.06[d] |
| **δ** | 2.019 | 84.171 | 70.83[f] |

[a]Ref. (Kvick *et al.*, 1980) [b] at 23 K. Ref. (Destro *et al.*, 2000) [c] (Perlovich *et al.*, 2001) [d] This work (100K), [e]This work (298K) [f] at 1.9 GPa. (Dawson *et al.*, 2005)

*S1.3 Structure solution and refinement of the neutron powder diffraction pattern obtained at 100 K*

All analysis was carried out using Topas-Academic (Coelho, 2015). The conventional space group setting of ζ-glycine is *P*1, and the position of the molecule in the unit cell is arbitrary; however, the coordinates for N1 were fixed at those determined in the structure prediction. To confirm that the predicted coordinates were correct the orientation of the glycine molecule were also determined by simulated annealing (the position is arbitrary in *P*1).

In order to emphasise its relationship with ε-glycine, the structure of ζ-glycine was transformed to a non-standard *I*1 setting for refinement. The transformation matrix for the unit cell basis vectors from the *P*1 to *I*1 setting is

$$\begin{pmatrix} -1 & 1 & 0 \\ 0 & -1 & 1 \\ 1 & 0 & 1 \end{pmatrix}.$$

The refinement model consisted of ζ- and ε-glycine, Pb (which was used as a pressure marker, (Fortes *et al.*, 2007, 2012)), $Al_2O_3$ and $ZrO_2$ (both components of the anvils of the Paris-Edinburgh press used to apply pressure). Glycine molecules were treated as variable-metric rigid bodies in which the bond distances and angles were fixed at those of γ-glycine, but the N1-C2-C1-O2 and C1-C2-N1-H3 torsion angles were allowed to vary; the other torsion angles involving H were assumed to be related to these by ±120°. In the final stages of refinement the two strongest lines of γ-glycine, corresponding to the 111 and 110 reflections, were introduced to account for two broad features at *d* = 2.96 and 3.47 Å. This approach was found to yield the same fitting statistics as an explicit model of γ-glycine, but at the cost of fewer parameters. The final Rietveld fit is shown in Fig. 2a of the main text. Crystal and refinement data are listed in Table S2.

*S1.4 The diffraction pattern at 290 K.*

A stack plot showing the changes in the diffraction pattern as the sample was warmed from 100 K to 290 K is shown in Fig. S11. The pattern at 290 K was modelled as a mixture of β-glycine with a small residue of the ζ-phase. The glycine phases were modelled using variable-metric rigid bodies, as described in Section S1.3 for the

100 K pattern. The two peaks assigned to γ-glycine were found to persist, and were also modelled in the same way as described above. Lead, $Al_2O_3$ and $ZrO_2$ were also included in the model. The final Rietveld fit is shown in Fig. 2b of the main paper. Crystal and refinement data are listed in Table S2.

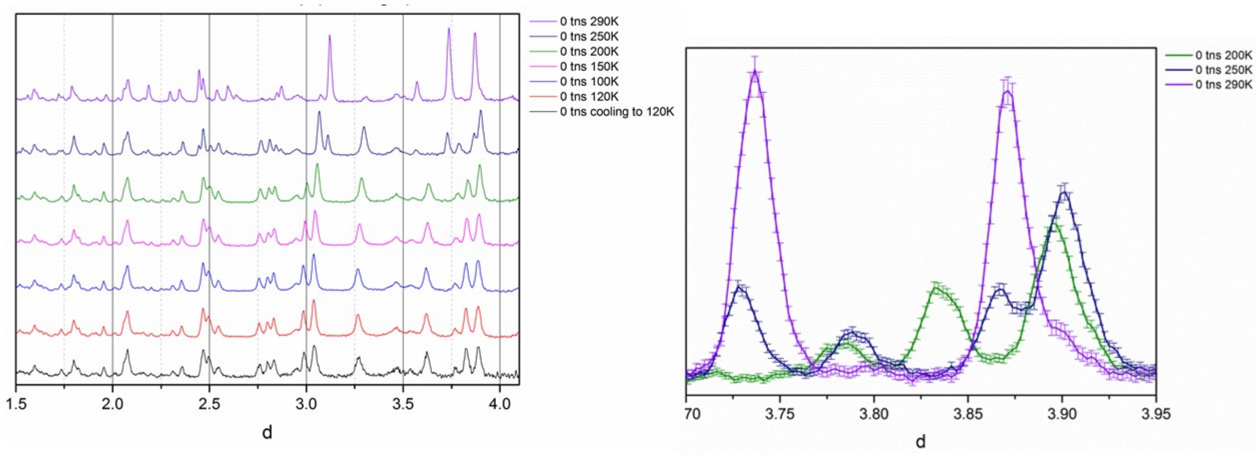


(a) (b)

**Figure S11**: (a) Diffraction data collected as the mixture of ε and ζ glycine obtained at 100 K was warmed to RT. Sample cooled to 120 K (black), stable at 120 K (red), stable at 100 K (blue), and then warmed in 50 K steps to 150K (magenta), 200 K (green), 250 K (navy) and 290 K (purple). (b) Expansion of the region between 3.7 and 3.95 Å at 200 K (green), 250 K (blue) and 290 K (purple).

*S1.5 PIXEL and symmetry adapted perturbation theory calculations.*

PIXEL and symmetry adapted perturbation theory enable the intermolecular interaction energies in a crystal structure to be estimated. For the PIXEL calculations, molecular electron densities were obtained using the program GAUSSIAN09 revision A.02 (M. J. Frisch, 2009) at the MP2 level with the 6-31G** basis set. The electron density was then analysed using the PIXELc module of CLP program package (Gavezzotti, 2011) which allows the calculation of dimer and lattice energies. Symmetry-adapted perturbation theory (SAPT) calculations (Jeziorski *et al.*, 1994, Stone, 2013) were carried out with the PSI4 code (Turney *et al.*, 2012) using the SAPT2+3 method with the aug-cc-pVDZ basis set. $\delta E_{HF}^{(3)}$ corrections were applied to induction energies in all cases (Hohenstein & Sherrill, 2010b, a, 2012).

Structures were visualised using XP (Sheldrick, 2001), MERCURY (Macrae *et al.*, 2008) and DIAMOND (CrystalImpact, 2004). PLATON was used for geometric analysis (Spek, 2003).

**Table S2**: Refinement data for refinement of the mixed ε/ζ and β/ζ phases at 100 and 290 K.

| Phase | ε | ζ | β | ζ |
|---|---|---|---|---|
| Temperature (K) | 100 | | 290 | |
| Load (Tonnes) | 0 | | | |
| Formula | $C_2D_5NO_2$ | | | |
| $M_r$ | 80.097 | | | |
| Crystal system | Monoclinic | Triclinic | Monoclinic | Triclinic |
| Space group | *Pn* | *I*1 | *P*2$_1$ | *I*1 |
| *a*, *b*, *c* (Å) | 5.0230(4)<br>5.9846(4)<br>5.4946(5) | 5.1000(4)<br>6.2850(3)<br>5.4295(3) | 5.0907(2)<br>6.25954(16)<br>5.38710(19) | 5.1029(16)<br>6.3450(12)<br>5.4331(18) |
| α, β, γ (°) | 90<br>114.654(8)<br>90 | 85.815(5)<br>114.456(5)<br>104.136(5) | 90<br>113.261(4)<br>90 | 85.91(3)<br>114.26(3)<br>103.55(3) |
| $V$ (Å$^3$) | 150.12(2) | 153.545(17) | 157.710(10) | 155.85(9) |
| $Z$ | 2 | | | |
| $D_x$ (Mg m$^{-3}$) | 1.772 | 1.732 | 1.686 | 1.707 |
| Radiation | Neutrons | | | |
| *d*-spacing range (Å) | 0.6 – 4.1 | | | |
| *R* factors (%) and goodness of fit | $R_p$ = 2.87<br>$R_{wp}$ = 3.09<br>$R_{exp}$ = 2.26<br>$S$ = 1.36 | | $R_p$ = 3.22<br>$R_{wp}$ = 3.55<br>$R_{exp}$ = 3.63<br>$S$ = 0.98 | |
| No. Parameters | 65 | | 62 | |
| Weighting scheme | Statistical ($w = 1/\sigma^2(I)$) | | | |
| Max(Δ/σ) | < 0.001 | | | |

## 2. Results and Discussion

### *2.1. Additional comments on the structure of ζ-Glycine:*

The first coordination spheres of ε and ζ-glycine each contain 14 molecules, and Fig. S12 shows the two structures in approximately equivalent orientations. The formation of layers in the ζ, ε and β phases is shown in Figs. S13-15. The structure of γ-glycine is shown in Fig. S16 for comparison. The interaction energies for ε and ζ-glycine are listed in Table S3; the values of the energies were calculated from the experimentally-determined structures of the ε- and ζ- phases reported here. Energies were calculated using both the PIXEL and SAPT2+3 methods, and the agreement in total energies between the two is excellent. The advantage of these methods,

aside from their accuracy, is that the total energies of the interactions are broken-down into electrostatic, polarisation, dispersion and repulsion contributions, which provides insight into the physical nature of each interaction. Although there are differences in the break-down of the different terms in the PIXEL and SAPT calculations because of the way the contributions are calculated, both calculations lead to the same conclusion: all interactions are dominated by the electrostatic term, with only a relatively modest contribution from dispersion.

In addition to the stabilising contacts described in the main text, the structure also exhibits some destabilising interactions. Within the layers there are relatively long interactions across the *ac* face diagonal of the unit cell in which the intermolecular energy is +16.0 kJ $mol^{-1}$. There are also some quite strongly repulsive (+43.3 kJ $mol^{-1}$) interlayer contacts featuring C2H2…O2 interactions. In both cases the source of the positive energy is a large positive electrostatic contribution which is not compensated for by either polarisation or dispersion. Overall six out of the fourteen interactions are repulsive or have essentially zero energy; this is similar to the situation described previously for the ε and γ phases, where 6/14 contacts are repulsive. The energies of molecules D and G and their equivalents in both phases remain largely unchanged through the transition, while those of E and F change from being respectively strongly repulsive and attractive, becoming much less energetic through attenuation of the electrostatic components.

ε

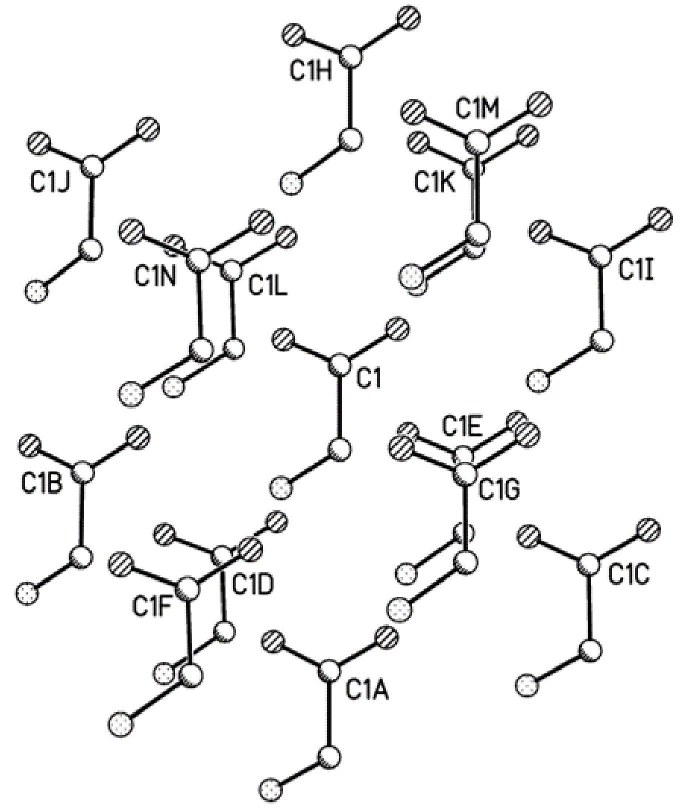


ζ

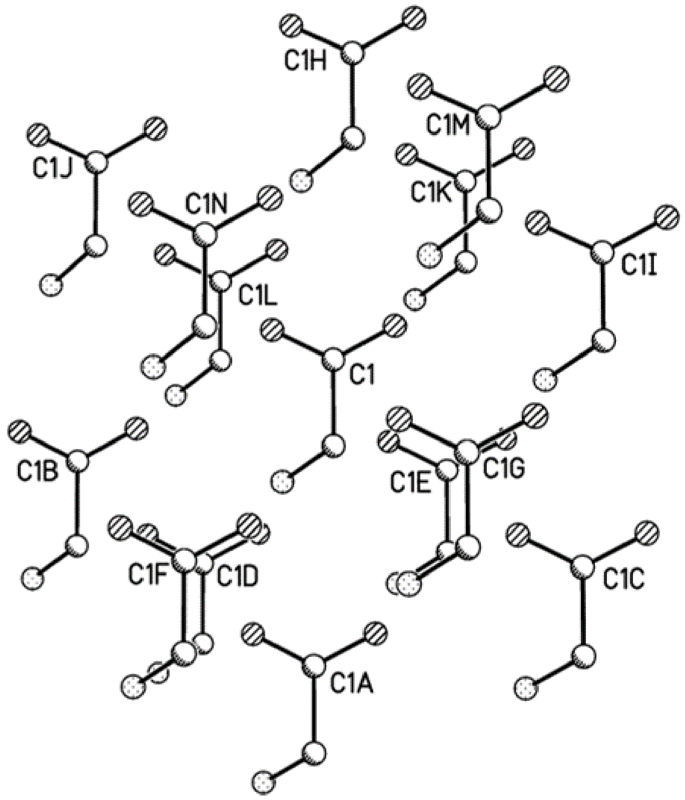


**Figure S12**: The first coordination spheres of ε and ζ glycine showing labelling used in Table S2.

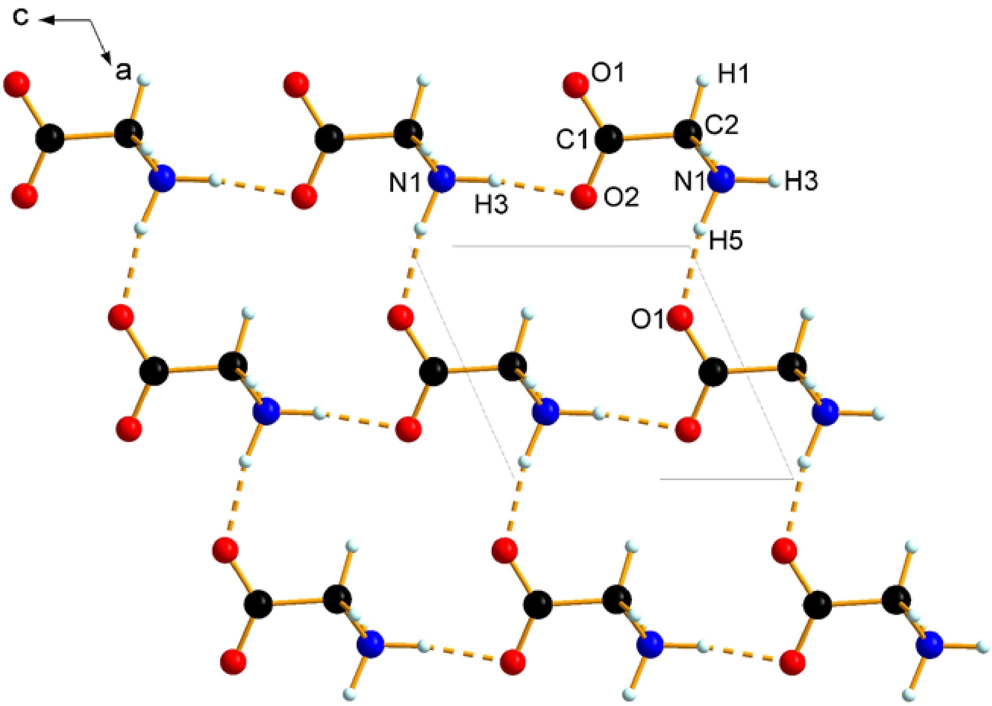


(*a*)

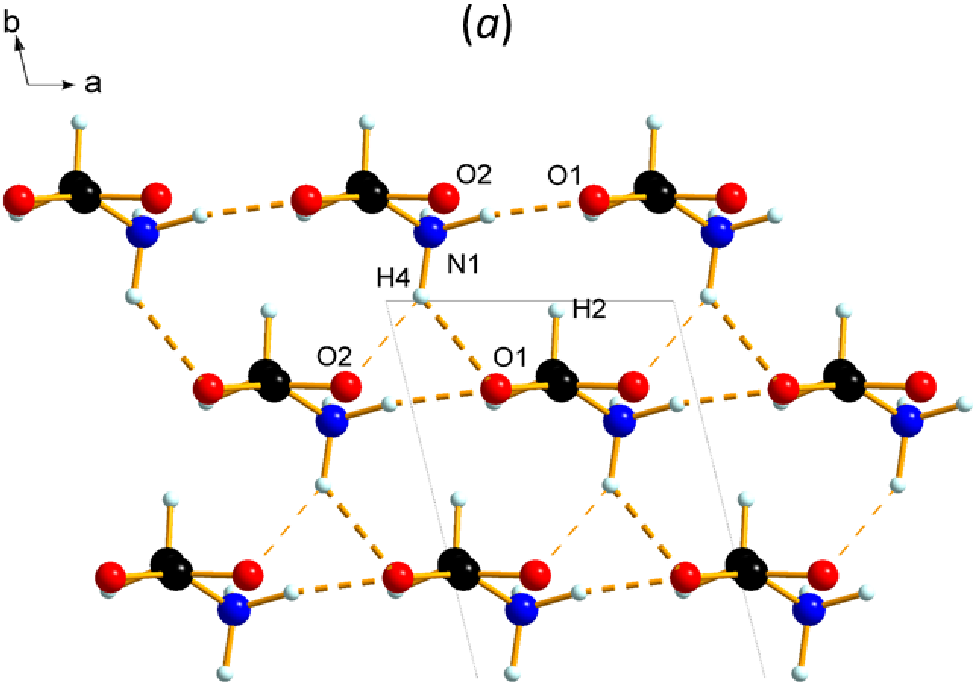


(*b*)

**Figure S13**: Intermolecular interactions in ζ-glycine. (a) Layers formed in in the *ac* plane viewed along **b**. (b) Stacking of the layers viewed along **c**.

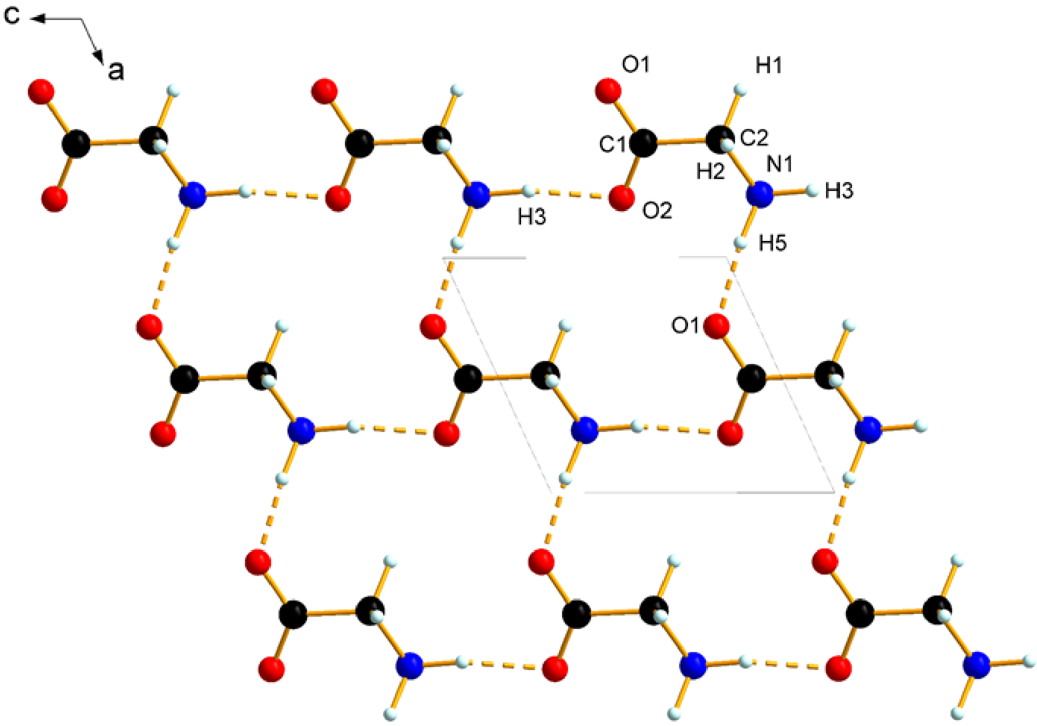


(a)

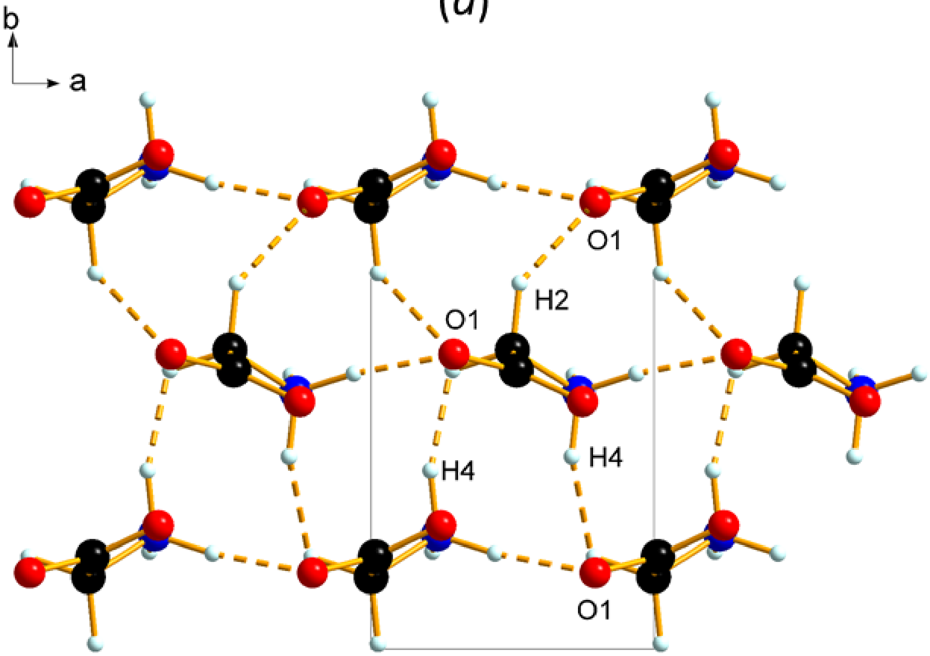


(b)

**Figure S14**: Intermolecular interactions in ε-glycine. (a) Layers formed in in the *ac* plane viewed along **b**. (b) Stacking of the layers viewed along **c**.

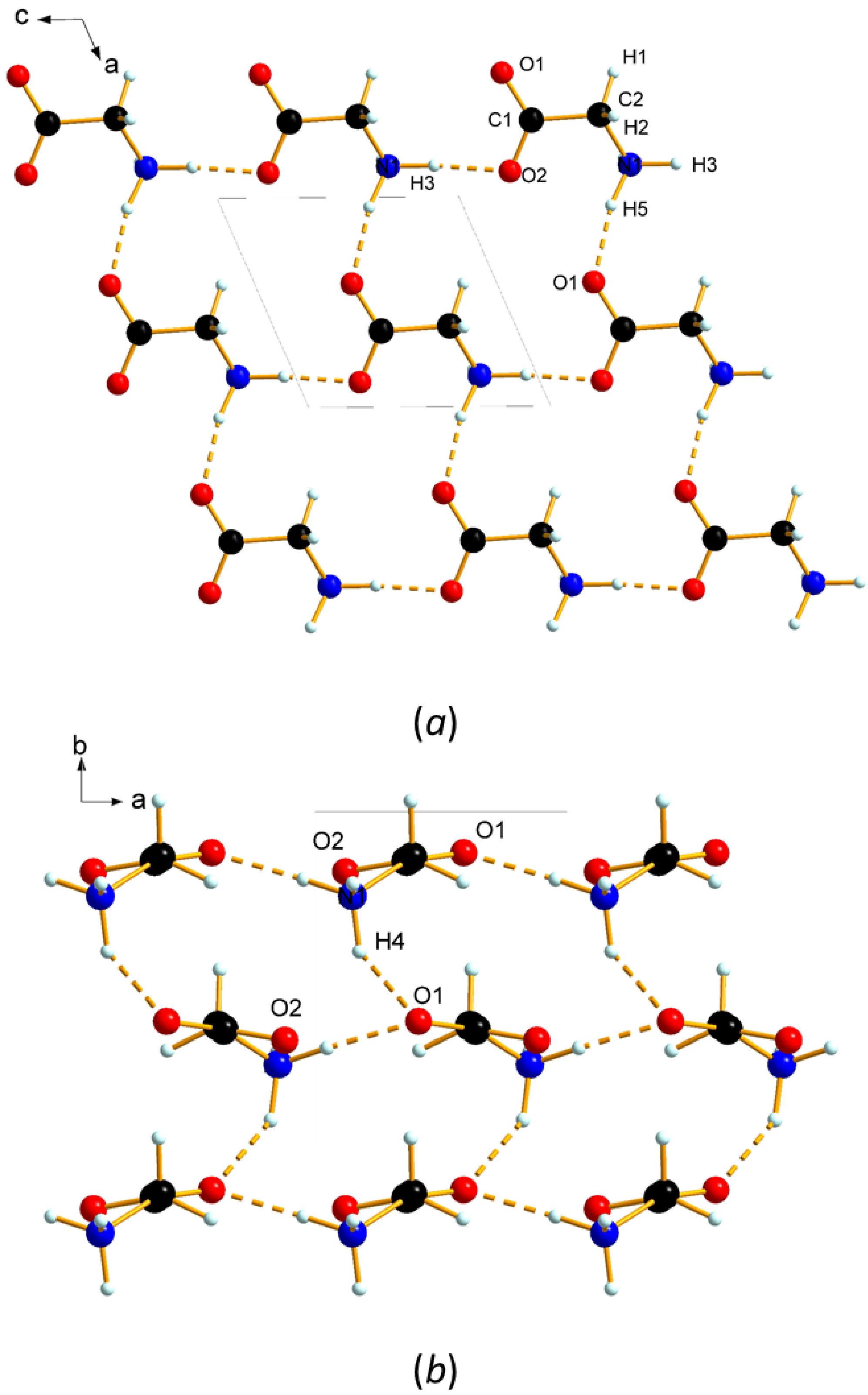


**Figure S15**: Intermolecular interactions in β-glycine. (a) Layers formed in in the *ac* plane viewed along **b**. (b) Stacking of the layers viewed along **c**.

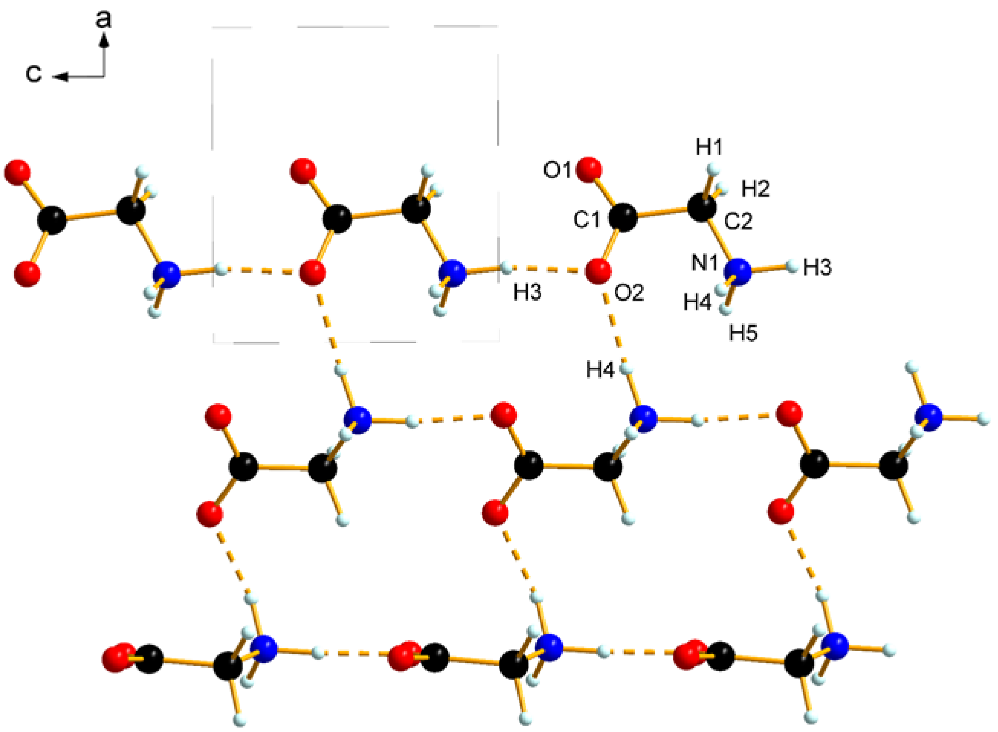


(a)

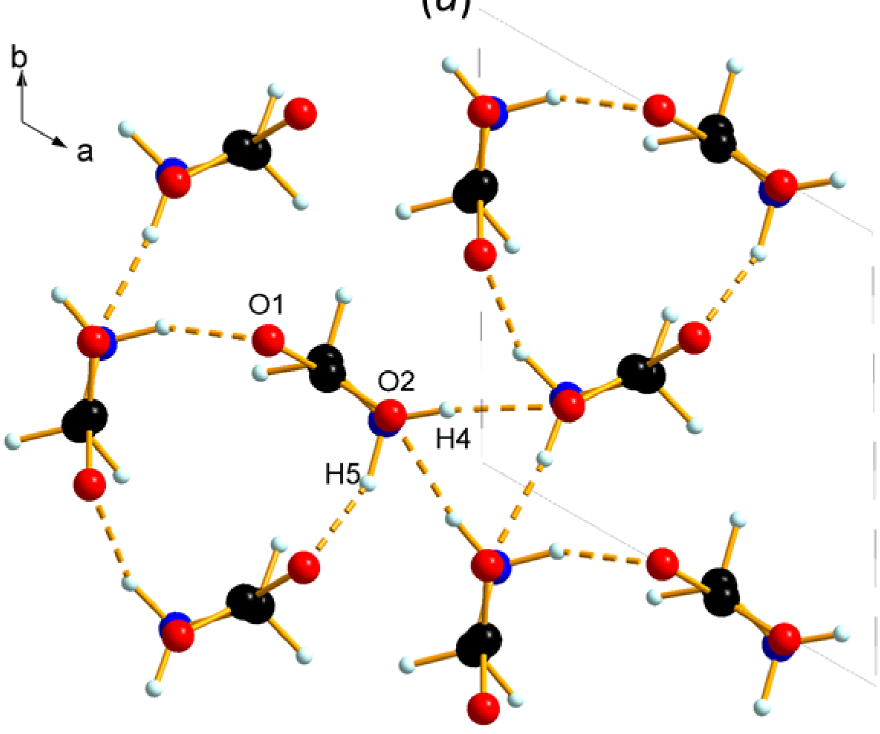


(b)

**Figure S16**: Intermolecular interactions in γ-glycine. The structure consists of a 3-dimensional network, rather than layers, but the fragments chosen here are intended for comparison with Figs S13-15 above. (a) View along **b**. (b) View along **c**. Coordinates taken from CSD entry GLYCIN16 and the structure inverted (Kvick *et al.*, 1980).

**Table S3**: Contact geometry and energies within the first coordination spheres in ε and ζ glycine. Energies are in kJ $mol^{-1}$, and the letters A…N refer to the labelling in Fig. S12; $d_{centroid}$ is the distance between the centroids of the molecules.

| ζ | | | | | ε | | | | |
|---|---|---|---|---|---|---|---|---|---|
| **# [$d_{centroid}$ (Å)]** | **Contact** | **Operation** | **H…O (Å)** | **<N/C-H…O (°)** | **#** | **Contact** | **Operation** | **H…O (Å)** | **<N/C-H…O (°)** |
| Contacts formed within layers | | | | | | | | | |
| **A/H** [5.429] | N1-H3...O1 | *x*, *y*, *z*-1 | 2.493(7) | 129.7(2) | **A/H** [5.495] | N1-H3...O1 | *x*, *y*, *z*-1 | 2.497(15) | 133.6(3) |
| | N1-H3...O2 | *x*, *y*, *z*-1 | 1.753(4) | 168.6(2) | | N1-H3...O2 | *x*, *y*, *z*-1 | 1.855(4) | 150.6(5) |
| PIXEL | $E_{elec}$ = -122.1 $E_{pol}$ = -40.3 $E_{disp}$ = -15.8 $E_{rep}$ = 70.4 **$E_{tot}$ =-107.8** | | | | PIXEL | $E_{elec}$ = -109.4 $E_{pol}$ = -32.7 $E_{disp}$ = -14.4 $E_{rep}$ = 54.0 **$E_{tot}$ =** -102.6 | | | |
| SAPT2+3 | $E_{elec}$ = -123.9 $E_{pol}$ = -47.5 $E_{disp}$ = -26.7 $E_{rep}$ = 89.2 **$E_{tot}$ =-108.9** | | | | SAPT2+3 | $E_{elec}$ = -112.0 $E_{pol}$ = -37.1 $E_{disp}$ = -23.6 $E_{rep}$ = 71.7 **$E_{tot}$ =** -101.1 | | | |
| **B/I** [5.100] | N1-H5...O1 | *x*+1, *y*, *z* | 1.775(9) | 166.8(9) | **B/I** [5.023] | N1-H5...O1 | *x*+1, *y*, *z* | 1.712(18) | 171.8(15) |
| PIXEL | $E_{elec}$ = -34.6 $E_{pol}$ = -36.5 $E_{disp}$ = -13.5 $E_{rep}$ = 58.8 **$E_{tot}$ = -25.8** | | | | PIXEL | $E_{elec}$ = -45.6 $E_{pol}$ = -42.3 $E_{disp}$ = -15.2 $E_{rep}$ = 69.7 **$E_{tot}$ =** -33.4 | | | |
| SAPT2+3 | $E_{elec}$ = -37.5 $E_{pol}$ = -42.6 $E_{disp}$ = -24.9 $E_{rep}$ = 78.6 **$E_{tot}$ = -26.5** | | | | SAPT2+3 | $E_{elec}$ = -48.8 $E_{pol}$ = -50.1 $E_{disp}$ = -27.3 $E_{rep}$ = 91.8 **$E_{tot}$ =** -34.5 | | | |
| **C/J** [5.706] | C2-H1...O2 | *x*-1, y,*z*-1 | 2.609(5) | 151.9(7) | **C/J** [5.692] | C2-H1...O2 | *x*-1, *y*, *z*-1 | 2.750(7) | 173.2(9) |
| PIXEL | $E_{elec}$ = 21.3 $E_{pol}$ = -4.5 $E_{disp}$ = -3.4 $E_{rep}$ = 2.6 **$E_{tot}$ = 16.0** | | | | PIXEL | $E_{elec}$ = 20.7 $E_{pol}$ = -3.7 $E_{disp}$ = -2.8 $E_{rep}$ = 1.4 **$E_{tot}$ =** 15.6 | | | |
| SAPT2+3 | $E_{elec}$ = 20.2 $E_{pol}$ = -4.8 $E_{disp}$ = -5.9 $E_{rep}$ = 4.9 **$E_{tot}$ = 14.4** | | | | SAPT2+3 | $E_{elec}$ = 19.9 $E_{pol}$ = -4.1 $E_{disp}$ = -4.8 $E_{rep}$ = 3.2 **$E_{tot}$ =** 14.2 | | | |
| Contacts formed between layers | | | | | | | | | |
| **D/M** [5.885] | N1-H4...O1 | *x*+½, *y*-½, *z*-½ | 2.274(5) | 121.1(10) | **D/K** [5.311] | N1-H4...O1 | *x*+½, -*y*+1, *z*-½ | 2.14(3) | 143.5(3) |
| PIXEL | $E_{elec}$ = -51.8 $E_{pol}$ = -11.5 $E_{disp}$ = -7.0 $E_{rep}$ = 10.9 **$E_{tot}$ = -59.4** | | | | PIXEL | $E_{elec}$ = -49.1 $E_{pol}$ =-17.1 $E_{disp}$ = -10.3 $E_{rep}$ = 16.3 **$E_{tot}$ =** -60.3 | | | |
| SAPT2+3 | $E_{elec}$ = -55.9 $E_{pol}$ = -13.4 $E_{disp}$ = -10.7 $E_{rep}$ = 20.7 **$E_{tot}$ = -59.4** | | | | SAPT2+3 | $E_{elec}$ = -51.4 $E_{pol}$ =-18.9 $E_{disp}$ = -14.8 $E_{rep}$ = 26.9 **$E_{tot}$ =** -58.2 | | | |
| **E/N** [3.917] | N1-H4...O2 | *x*-½, *y*-½, *z*-½ | 2.027(13) | 146.3(7) | **E/L** [4.086] | N1-H4...O2 | *x*-½, -*y*+1, *z*-½ | 2.542(14) | 123.8(9) |
| | | | | | | C2-H1...O2 | *x*-½, -*y*+1, *z*-½ | 2.59(3) | 105.5(4) |
| PIXEL | $E_{elec}$ = 18.9 $E_{pol}$ = -29.7 $E_{disp}$ = -19.4 $E_{rep}$ = 29.0 **$E_{tot}$ = -1.1** | | | | PIXEL | $E_{elec}$ = 60.2 $E_{pol}$ =-22.1 $E_{disp}$ = -15.7 $E_{rep}$ = 14.6 **$E_{tot}$ =** 36.9 | | | |
| SAPT2+3 | $E_{elec}$ = 9.9 $E_{pol}$ = -32.5 $E_{disp}$ = -28.5 $E_{rep}$ = 52.1 **$E_{tot}$ = 1.0** | | | | SAPT2+3 | $E_{elec}$ = 52.4 $E_{pol}$ =-20.0 $E_{disp}$ = -22.8 $E_{rep}$ = 30.8 **$E_{tot}$ =** 40.3 | | | |
| **F/K** [4.932] | C2-H2...O1 | *x*+½, *y*+½, *z*-½ | 2.181(7) | 158.4(7) | **F/M** [5.379] | C2-H2...O1 | *x*+½, -*y*+2, *z*-½ | 2.33(3) | 133.4(4) |
| PIXEL | $E_{elec}$ = -3.7 $E_{pol}$ = -13.2 $E_{disp}$ = -8.9 $E_{rep}$ = 17.4 **$E_{tot}$ = -8.3** | | | | PIXEL | $E_{elec}$ = -28.6 $E_{pol}$ =-12.1 $E_{disp}$ = -8.7 $E_{rep}$ = 12.7 **$E_{tot}$ =** -36.6 | | | |
| SAPT2+3 | $E_{elec}$ = -6.8 $E_{pol}$ = -13.7 $E_{disp}$ = -14.2 $E_{rep}$ = 25.8 **$E_{tot}$ = -9.0** | | | | SAPT2+3 | $E_{elec}$ = -34.1 $E_{pol}$ =-12.8 $E_{disp}$ = -13.4 $E_{rep}$ = 21.7 **$E_{tot}$ =** -38.6 | | | |
| **G/L** [4.548] | C2-H2...O2 | *x*-½, *y*+½, *z*-½ | 2.699(5) | 123.0(6) | **G/N** [4.173] | C2-H2...O2 | *x*-½, -*y*+2, *z*-½ | 2.456(15) | 139.8(12) |
| PIXEL | $E_{elec}$ = 57.9 $E_{pol}$ = -10.9 $E_{disp}$ = -9.5 $E_{rep}$ = 5.8 **$E_{tot}$ = 43.3** | | | | PIXEL | $E_{elec}$ = 54.4 $E_{pol}$ =-12.0 $E_{disp}$ = -13.5 $E_{rep}$ = 14.0 **$E_{tot}$ =** 42.9 | | | |
| SAPT2+3 | $E_{elec}$ = 57.3 $E_{pol}$ = -10.2 $E_{disp}$ = -13.5 $E_{rep}$ = 14.1 **$E_{tot}$ = 47.7** | | | | SAPT2+3 | $E_{elec}$ = 53.5 $E_{pol}$ =-12.3 $E_{disp}$ = -19.1 $E_{rep}$ = 23.7 **$E_{tot}$ =** 45.8 | | | |

*S2.2 Positions of the molecules in β, ε and ζ glycine.*

The positions of the centroid of the molecules in the unit cells of β, ε and ζ glycine, which all have $Z$ = 2, are listed in Table S4. The space groups, $P2_1$, $Pn$ and $I1$ are polar, and the unit cell origin may be chosen arbitrarily along the polar directions. In addition, unit cell translations of ½ are possible along the $a$ and $c$ directions of $P2_1$, these being elements of the Euclidean normaliser of this space group. The transformed centroid positions to near [¼, ¼, ¼] are also shown in Table S3.

**Table S4**: Positions of molecules in β, ε and ζ glycine

| | Centroid coordinates as refined | | | Centroid transformed to near ¼ ¼ ¼ | | | |
|---|---|---|---|---|---|---|---|
| | x | y | z | x | y | z | Distance (Å) from ¼ ¼ ¼ |
| β | 0.715 | 0.316 | 0.346 | 0.215 | 0.25 | 0.346 | 0.61 |
| ε | 0.688 | 0.745 | 0.677 | 0.25 | 0.245 | 0.25 | 0.04 |
| ζ | 0.601 | 0.746 | 0.895 | 0.25 | 0.25 | 0.25 | 0 |

**References**


Coelho, A. (2015). *TOPAS-Academic: General Profile and Structure Analysis Software for Powder Diffraction Data. Version 5.*

CrystalImpact (2004). *DIAMOND version 3.0, Visual crystal structure information system*

Dawson, A., Allan, D. R., Belmonte, S. A., Clark, S. J., David, W. I., McGregor, P. A., Parsons, S., Pulham, C. R. & Sawyer, L. (2005). *Crystal growth & design* **5**, 1415-1427.

Destro, R., Roversi, P., Barzaghi, M. & Marsh, R. E. (2000). *The Journal of Physical Chemistry A* **104**, 1047-1054.

Fortes, A. D., Wood, I. G., Alfredsson, M., Vocadlo, L., Knight, K. S., Marshall, W. G., Tucker, M. G. & Fernandez-Alonso, F. (2007). *High Pressure Res.* **27**, 201-212.

Fortes, A. D., Wood, I. G., Alfredsson, M., Vocadlo, L., Knight, K. S., Marshall, W. G., Tucker, M. G. & Fernandez-Alonso, F. (2012). *High Pressure Research* **32**, 337-337.

Gavezzotti, A. (2011). *New Journal of Chemistry* **35**, 1360-1368.

Hohenstein, E. G. & Sherrill, C. D. (2010a). *Journal of Chemical Physics* **133**, 014101/014101-014101/014112.

Hohenstein, E. G. & Sherrill, C. D. (2010b). *Journal of Chemical Physics* **133**, 104107/104101-104107/104107.

Hohenstein, E. G. & Sherrill, C. D. (2012). *Wiley Interdisciplinary Reviews: Computational Molecular Science* **2**, 304-326.

Jeziorski, B., Moszynski, R. & Szalewicz, K. (1994). *Chemical Reviews (Washington, DC, United States)* **94**, 1887-1930.

Kvick, A., Canning, W. M., Koetzle, T. F. & Williams, G. J. B. (1980). *Acta Crystallographica, Section B: Structural Science, Crystal Engineering and Materials* **B36**, 115-120.

M. J. Frisch, G. W. T., H. B. Schlegel, G. E. Scuseria, M. A. Robb, J. R. Cheeseman, G. Scalmani, V. Barone, B. Mennucci, G. A. Petersson, H. Nakatsuji, M. Caricato, X. Li, H. P. Hratchian, A. F. Izmaylov, J. Bloino, G. Zheng, J. L. Sonnenberg, M. Hada, M. Ehara, K. Toyota, R. Fukuda, J. Hasegawa, M. Ishida, T. Nakajima, Y. Honda, O. Kitao, H. Nakai, T. Vreven, J. A. Montgomery, Jr., J. E. Peralta, F. Ogliaro, M. Bearpark, J. J. Heyd, E. Brothers, K. N. Kudin, V. N. Staroverov, R. Kobayashi, J. Normand, K. Raghavachari, A. Rendell, J. C. Burant, S. S. Iyengar, J. Tomasi, M. Cossi, N. Rega, J. M. Millam, M. Klene, J. E. Knox, J. B. Cross, V. Bakken, C. Adamo, J. Jaramillo, R. Gomperts, R. E. Stratmann, O. Yazyev, A. J. Austin, R. Cammi, C. Pomelli, J. W. Ochterski, R. L. Martin, K. Morokuma, V. G. Zakrzewski, G. A. Voth, P. Salvador, J. J. Dannenberg, S. Dapprich, A. D. Daniels, Ö. Farkas, J. B. Foresman, J. V. Ortiz, J. Cioslowski, and D. J. Fox (2009). *Gaussian 09.*

Macrae, C. F., Bruno, I. J., Chisholm, J. A., Edgington, P. R., McCabe, P., Pidcock, E., Rodriguez-Monge, L., Taylor, R., van de Streek, J. & Wood, P. A. (2008). *J. Appl. Cryst.* **41**, 466-470.

Perlovich, G., Hansen, L. & Bauer-Brandl, A. (2001). *Journal of Thermal Analysis and Calorimetry* **66**, 699-715.

Sheldrick, G. M. (2001). *SHELXTL-XP version 6.01.*

Spek, A. L. (2003). *J. Appl. Cryst.* **36**, 7-13.

Stone, A. J. (2013). *The Theory of Intermolecular Forces*. Oxford: Oxford University Press.

Turney, J. M., Simmonett, A. C., Parrish, R. M., Hohenstein, E. G., Evangelista, F. A., Fermann, J. T., Mintz, B. J., Burns, L. A., Wilke, J. J., Abrams, M. L., Russ, N. J., Leininger, M. L., Janssen, C. L., Seidl, E. T., Allen, W. D., Schaefer, H. F., King, R. A., Valeev, E. F., Sherrill, C. D. & Crawford, T. D. (2012). *Wiley Interdisciplinary Reviews: Computational Molecular Science* **2**, 556-565.
