# Supplementary figures and images for: ζ-Glycine: insight into the mechanism of a polymorphic phase transition
Source: IUCrJ. 2017 Sep 1;4(Pt 5):569–74. doi: 10.1107/S205225251701096X (PMC5619850; doi:10.1107/S205225251701096X)

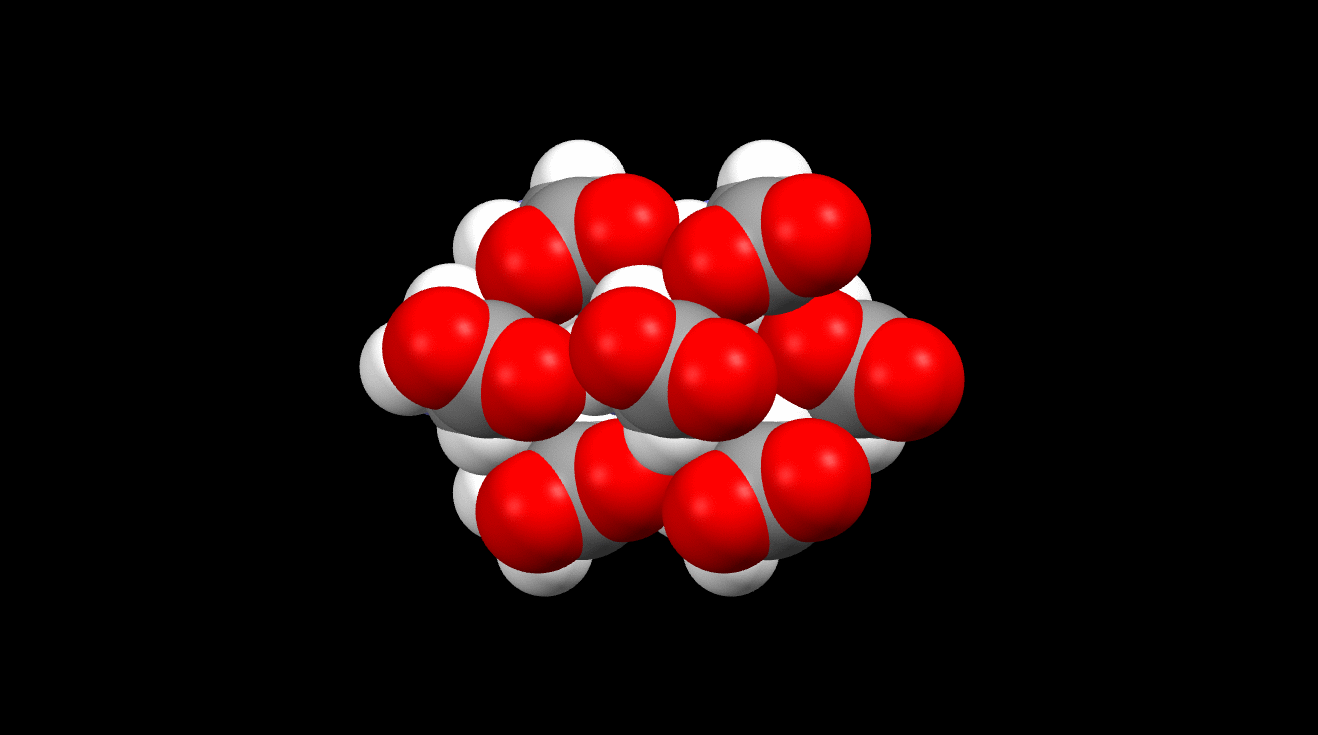

Supplement: Supplementary file 3 [file m-04-00569-sup3.gif]
